# *Rho*, a Fraction From *Rhodiola crenulate*, Ameliorates Hepatic Steatosis in Mice Models

**DOI:** 10.3389/fphys.2018.00222

**Published:** 2018-03-14

**Authors:** Qin Yi, Puyang Sun, Juan Li, Siming Kong, Jinying Tian, Xuechen Li, Yanan Yang, Peicheng Zhang, Yuying Liu, Jingyan Han, Xiaolin Zhang, Fei Ye

**Affiliations:** ^1^Beijing Key Laboratory of New Drug Mechanisms and Pharmacological Evaluation Study, Institute of Materia Medica, Chinese Academy of Medical Science & Peking Union Medical College, Beijing, China; ^2^State Key Laboratory of Bioactive Substance and Function of Natural Medicines, Institute of Materia Medica, Chinese Academy of Medical Science & Peking Union Medical College, Beijing, China; ^3^Tasly Microcirculation Research Center, Peking University Health Science Center, Beijing, China

**Keywords:** a fraction from *Rhodiola crenulate* (*Rho*), non-alcoholic fatty liver disease (NAFLD), hepatic steatosis, insulin resistance, hepatic lipid metabolism

## Abstract

The prevalence of non-alcoholic fatty liver disease (NAFLD), which is developed from hepatic steatosis, is increasing worldwide. However, no specific drugs for NAFLD have been approved yet. To observe the effects of *Rho*, a fraction from *Rhodiola crenulate*, on non-alcoholic hepatic steatosis, three mouse models with characteristics of NAFLD were used including high-fat diet (HFD)-induced obesity (DIO) mice, KKAy mice, and HFD combined with tetracycline stimulated Model-T mice. Hepatic lipid accumulation was determined via histopathological analysis and/or hepatic TG determination. The responses to insulin were evaluated by insulin tolerance test (ITT), glucose tolerance test (GTT), and hyperinsulinemic-euglycemic clamp, respectively. The pathways involved in hepatic lipid metabolism were observed via western-blot. Furthermore, the liver microcirculation was observed by inverted microscopy. The HPLC analysis indicated that the main components of *Rho* were flavan polymers. The results of histopathological analysis showed that *Rho* could ameliorate hepatic steatosis in DIO, KKAy, and Model-T hepatic steatosis mouse models, respectively. After *Rho* treatment in DIO mice, insulin resistance was improved with increasing glucose infusion rate (GIR) in hyperinsulinemic-euglycemic clamp, and decreasing areas under the blood glucose-time curve (AUC) in both ITT and GTT; the pathways involved in fatty acid uptake and *de novo* lipogenesis were both down-regulated, respectively. However, the pathways involved in beta-oxidation and VLDL-export on hepatic steatosis were not changed significantly. The liver microcirculation disturbances were also improved by *Rho* in DIO mice. These results suggest that *Rho* is a lead nature product for hepatic steatosis treatment. The mechanism is related to enhancing insulin sensitivity, suppressing fatty acid uptake and inhibiting *de novo* lipogenesis in liver.

## Introduction

Non-alcoholic fatty liver disease (NAFLD), a spectrum of liver disease developing progressively from simple steatosis to steatohepatitis, fibrosis, and cirrhosis, is increasing worldwide with the incidence of 20–30% in Western countries and 5–18% in Asia (Sayiner et al., 2016) and is caused by a multitude of factors including the insulin resistance (Bugianesi et al., 2010; Petta et al., 2016). In normal conditions, insulin inhibits the lipolysis of white adipose tissue and gluconeogenesis of liver, but promotes hepatic lipogenesis; in insulin-resistant states, usually, the inhibitions of lipolysis and gluconeogenesis are failed, but the promotion of lipogenesis is preserved (Gruben et al., 2014). As a result, the auxo-action of insulin resistance in hepatic steatosis/NASH is induced by enhancing *de novo* lipogenesis in liver and subsequent lipolysis in adipose tissue. Therefore, insulin resistance plays a key role in the progression of NAFLD and maybe a therapeutic target for its treatment.

Vitamin E and pioglitazone currently remain the first line off label drugs for NASH. Several agents are currently in intermediate or advanced stages of development, however, no specific drugs for NAFLD have been approved yet (Banini and Sanyal, 2017). Here, we identified *Rho*, the active fractions from *Rhodiola crenulate*, as an effective nature product for NAFLD treatment. *Rhodiola crenulata* has been used for the treatment of cardiovascular disease for more than one thousand years in China. It is classified to *Crassulaceae* family, and contains phenols, flavonoids, and other compounds, which produce antioxidant, anti-inflammatory, antidepressant, anti-fatigue, cardiac protection, neuroprotection, and anti-lipogenesis effects (Wu et al., 2009; Chan, 2012; Grech-Baran et al., 2015). In this study, we demonstrated that *Rho* could improve hepatic steatosis in three NAFLD animal models. Moreover, its mechanism, including the insulin sensitizing effect and the pathways involved in hepatic lipid metabolism, was investigated in DIO mice.

## Methods

### Preparation of active fractions from *Rhodiola crenulata* (*Rho*)

The roots of *R. crenulata* were collected in October 2010 from Shannan Tibet Autonomous Region, and identified by Prof. Lin Ma (Institute of Materia Medica, Peking Union Medical College and Chinese Academy of Medical Sciences, Beijing, China). Its voucher specimen was deposited in the Herbarium of the Department of Medicinal plants, Institute of Materia Media, Chinese Academy of Medical Sciences and Peking Union Medical College, Beijing, P. R. China.

Dried roots (100 kg) of *R. crenulata* were extracted with 80% EtOH for three times. After the solvent was evaporated under reduced pressure, the residue was resuspended in H_2_O (50 L) for 24 h. The deposits were filtered from the solution. Then, the aqueous layer (18 Kg) was concentrated under reduced pressure, and applied to a HP-20 macroporous adsorbent resin (40 Kg, dried weight) column. Successive elution from the column with H_2_O (RC-1), 15% EtOH (RC-2), 30% EtOH (RC-3), 50% EtOH (RC-4), 70% EtOH (RC-5), and finally 95% EtOH (RC-6) yielded five corresponding fractions after removing solvents.

The chemical constituents of the prepared RC-3 were analyzed by high-performance liquid chromatography (HPLC) consisting of a quaternary delivery system, an auto-sampler, and a diode array detector (DAD). The analysis was performed on an Apollo C18 (250 mm × 4.6 mm, 5 μm) column using the mixture of methanol (A) and 0.2% acetic acid (B) as mobile phase in an elution program (0–30 min, 5% A to 15% A; 30–60 min, 15% A; 60–80 min, 15% A to 25% A; 80–90 min, 25% A to 100% A; 90–100 min, 100% A). The flow rate was 1.0 ml/min and the chromatogram was recorded at 280 nm.

### Chemicals and reagents

Fenofibrate (Feno) from Fournier Pharma (France); Fluvastatin (Flu) from Beijing Novartis Pharma Ltd; Tetracycline was purchased from AMRESCO (USA); Polyene Phosphatidylcholine (PPC) from Sanofi, Beijing (China); Rosiglitazone (Rosi) from GSK, China; Anti-β-actin antibody, anti-FAS antibody, anti-ACC antibody from Cell Signaling Technology (USA); anti-MTTP antibody, anti-CPT-1 antibody, anti-CD36 antibody from Abcam (USA); anti-SREBP-1c antibody from SANTA (USA); commercial kits for liver triglyceride, ECL reactions from Applygen Technologies Inc.

### Animal and diets

All animals were purchased from the Animal Center of Institute of Laboratory Animal Sciences, CAMS & PUMC, and grouped housed (four mice per cage) in grommet cages under the condition of temperature 21~23°C, humidity 40~60%, 12 h light/dark cycle, *ad libitum* access to water and chow diet. Efforts were made to minimize animal suffering. All animal experiments were performed in accordance with the guidelines established by the National Institutes of Health for the care and use of laboratory animals and were approved by the Animal Care Committee of CAMS & PUMC.

The DIO mice were induced by HFD (containing 50% fat, 36% carbohydrate, and 14% protein in energy) in male 4 weeks C57BL/6 mice. After a 12-week induction, mice with body weight >40 g were selected and randomly divided into three groups (*n* = 8): the DIO group, Positive drug group (Feno, 100 mg/kg body weight/d; or Flu, 50 mg/kg body weight/d; or Rosi, 8 mg/kg body weight/d), and *Rho* treatment group (200 mg/kg body weight/d). Meanwhile, aged-matched mice fed with the standard chow diet (containing 12% fat, 62% carbohydrate, and 26% protein in energy) were used as normal control (Con).

The male KKAy mice, which were 11 weeks old with the average weight about 35 g, were fed chow diet (1K65, Beijing HFK Bioscience Co. Ltd., China), and randomly divided into three groups (*n* = 8): model control KKAy, PPC-K, and *Rho*-K; and orally treated with water, PPC (200 mg/kg body weight/d), and *Rho* (400 mg/kg body weight/d), respectively.

Model-T mice were induced with HFD for 11 weeks and then intraperitoneally injected with tetracycline (50 mg/kg body weight/d) for 17 days in male 4 weeks old C57BL/6 mice. The model mice were also divided into three groups (*n* = 8): the model control Model-T, PPC-T, and *Rho*-T; administrated with water, PPC (200 mg/kg body weight/d), and *Rho* (200 mg/kg body weight/d), respectively. Aged-matched mice fed with the standard chow diet were used as normal control (Con-T).

### Insulin sensitivity evaluation

Insulin tolerance test (ITT) and intraperitoneal glucose tolerance test (IPGTT) were performed as previously described (Tian et al., 2015). Briefly, mice were fasted for 2 h. Blood samples were collected from tails for determination of baseline values of blood glucose (*t* = 0 min). The mice were then subcutaneous injected with insulin 0.27 U/kg or intraperitoneal injected with glucose 1 g/kg, then blood samples were collected at 30, 60, 120 min for glucose measurement. The levels of blood glucose were measured by a glucose oxidase (GOD) method. The values of area under the glucose-time curve (AUC) were calculated.

The hyperinsulinemic-euglycemic clamp test was conducted according to a protocol previously published (Ye et al., 2008). Briefly, after fasting for 4 h, the serum insulin level of mouse was clamped by injecting insulin at 60 pmol/kg/min rates, meanwhile, its homeostatic blood glucose was maintain at normal physiological level by infusing 10% glucose at the different rates. The glucose infusion rate (GIR) was calculated when the blood glucose was maintained at 95 ± 5 mg/dl for more than 20 min.

### Histological analysis of liver

After 5 weeks of treatment for DIO mice, 8 weeks of treatment for KKAy mice, 6 weeks of treatment for Model-T mice, mice were sacrificed after a 16-h fasting. Liver tissues were dissected quickly on ice. Parts of them (the left middle lobe) were immediately fixed in 4% paraformaldehyde, paraffin embedded, and then stained with hematoxylin and eosin. Hepatic steatosis was graded on the basis of the semiquantitative scoring system as previously described (Ma et al., 2011).

### Determination of triglyceride content in liver

After 5 weeks of treatment for DIO mice, TG content were determined, the Left lateral lobe was collected. The TG content of liver were extracted according to the method published previously (Bligh and Dyer, 1959), and determined with the commercial biochemical kits (Jian Cheng Bioengineering Institute, Nanjing, China).

### Western-blot analysis

After 5 weeks of treatment for DIO mice, livers were homogenized in ice-cold buffer (containing 50 mM HEPES, 10 mM sodium pyrophosphate, 100 mM sodium fluoride, 2 mM sodium orthovanadate, 1% NP-40, 4 mM EDTA, and 2 mM PMSF, pH 7.4). Western blotting was performed to determine the pathways related to hepatic lipid metabolism as previously described (Ma et al., 2011). The gel image analysis system (Flurochem 5500, Alpha Innotech, USA) was used for the images acquisition.

### Determination of microcirculatory parameters

The hepatic microcirculation in DIO mice was observed with stereomicroscope (DM-IRB, Leica, Germany), and recorded with a color camera (JK-TU53H, 3CCD camera, Toshiba, Japan) and a DVD recorder (DVR-R25, Malata, China), respectively, as the method previously described (Chen et al., 2008).

### Statistical methods

The data were analyzed by one-way ANOVA analysis. All values are presented as means ± *SD*, and statistical significance was set at a value of *P* < 0.05.

## Results

### Analysis of chemical constituents in *Rho*

**Figure 1:**
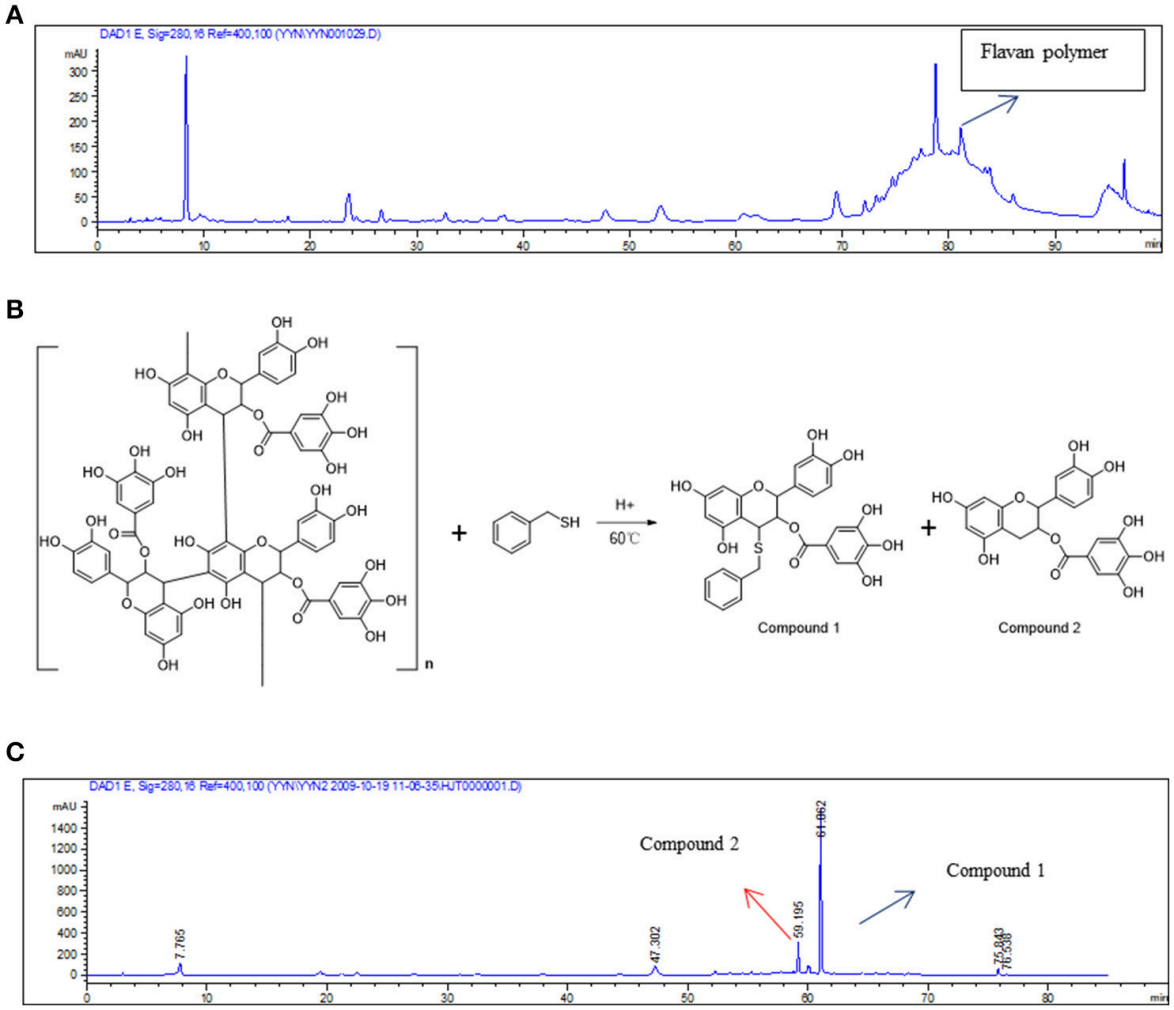
Analysis of chemical constituents in RC-3. **(A)** HPLC analysis of RC-3. **(B)** Acid-mediated depolymerization of flavan polymers in the presence of thiol nucleophiles. **(C)** HPLC analysis of the thiolysis of the RC-3.

The results indicated that the main components of the RC-3 are flavan polymers (Figure 1A). A literature (Thompson et al., 1972) search revealed that acid-mediated depolymerization of flavan polymers in the presence of thiol nucleophiles leads to β-C-4 substituted flavanol derivatives (Figure 1B). Hence, the main contents of RC-3 were confirmed as flavan polymers by using the reported method. The analysis of the thiolysis of RC-3 was performed by the same HPLC method with that of RC-3 (Figure 1C)

### *Rho* ameliorates hepatic steatosis in 3 animal models

**Figure 2:**
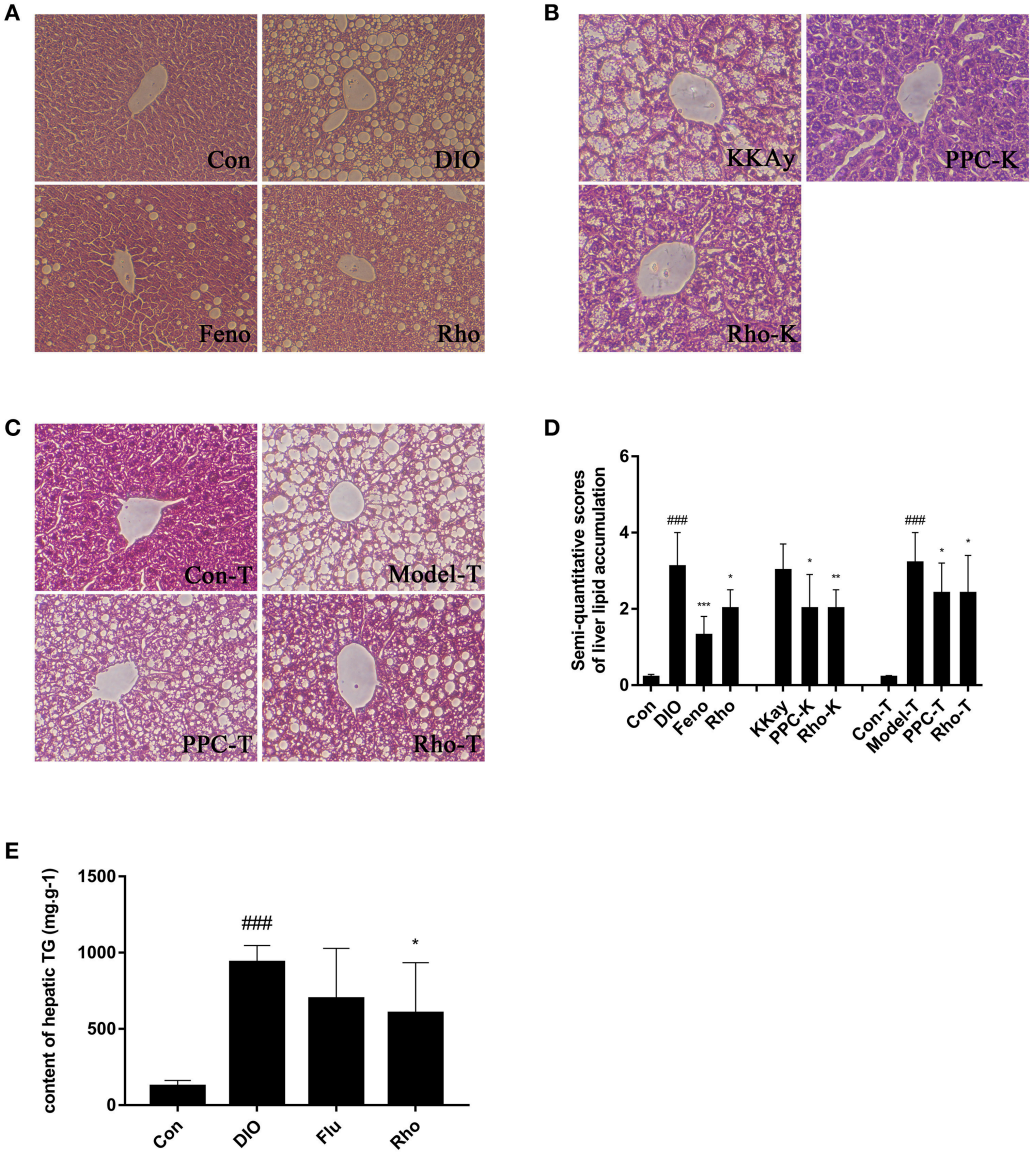
The effect of *Rho* on hepatic steatosis. **(A)** Liver histopathological analysis in DIO mice. **(B)** Liver histopathological analysis in KKAy mice. **(C)** Liver histopathological analysis in high-fat diet combined with tetracycline-induced Model-T mice. (H&E stained) **(D)** Semiquantitative scoring of steatosis. **(E)** Hepatic triglyceride content. ^###^*p* < 0.001 vs. normal control group; ^*^p < 0.05, ^**^p < 0.01, ^***^p < 0.001, vs. model control group, respectively, *n* = 8.

The hepatic steatosis in DIO mice was induced by long-term *ad libitum* feeding of HFD which contents much higher fat (50%) compared with the chow diet (12%). In this manner, the process of hepatic steatosis reflects well the clinical cases (Cong et al., 2008). The DIO mice displayed marked macrovesicular and microvesicular steatosis in liver (Figure 2A). Semiquantitative scoring result showed that the score of the DIO mice was 2.1-fold increased compared with that of Con mice (Figure 2D). The hepatic steatosis was significantly improved by *Rho* treatment with 35.5% reduction in lipid accumulation compared with DIO mice (Figures 2A,D). The hepatic TG content in DIO mice was 6.5-fold increased compared with that in Con mice. Notably, the elevated TG content was decreased by 35.8% in *Rho* treatment group (Figure 2E).

The KKAy mice are tipical T2DM animal model with obese, hyperglycemic, hyperinsulinemic, insulin resistant, and obvious hepatic steatosis (Yamamoto et al., 2010). KKAy mice exhibited obvious hepatic steatosis with hepatocyte lipidosis throughout the entire lobule, and the hepatic cords structure was not clear (Figure 2B). After 8 weeks *Rho* treatment, the elevated hepatocyte lipidosis was completely ameliorated with 33.3% decrease in the hepatic steatosis scores compared with that in KKAy mice (Figures 2B,D).

Model-T is an animal model of drug-induced hepatotoxicity and steatosis (Heaton et al., 2007; Brüning et al., 2014). It is found that tetracycline can induce hepatic mcrovesicular steatosis, which is severe and even fatal in some vulnerable patients, by regulating the expressions of the genes associated with lipid metabolism, such as increased biosynthesis of TG and cholesterol, decreased β-oxidation of fatty acids (Bhagavan et al., 1982; Yin et al., 2006). In Model-T mice, the lobules displayed severe lipidosis and ballooning degeneration (Figure 2C). *Rho* treatment significantly ameliorated the steatosis and hepatocyte swelling. The steatosis score in Model-T group was significantly increased compared with that in Con-T group, and decreased by 25% after *Rho* treatment (Figure 2D).

### *Rho* improves insulin resistance in DIO mice

**Figure 3:**
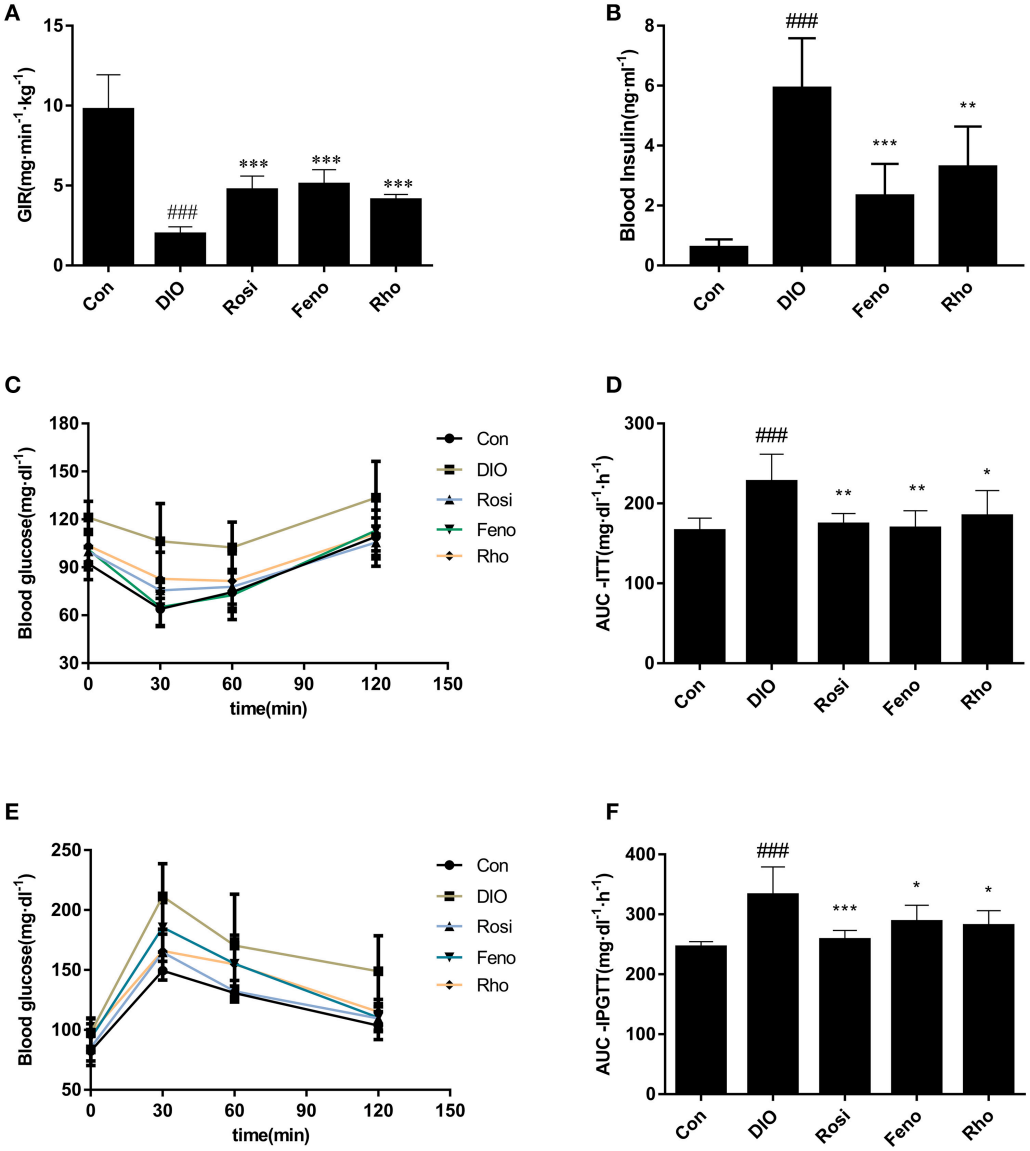
Effects of *Rho* on insulin resistance in high-fat-diet induced DIO mice. **(A)** Values of GIR in hyperinsulinemic-euglycemic clamp test. The DIO mice were administrated with *Rho* (200 mg/kg) for 20 days. Both rosiglitazone (Rosi, 10 mg/kg) and fenofibrate (Feno, 10 mg/kg) were used as the positive control. After fasting for 4 h, the animals were infused insulin at 60 pmol/kg/min rates and 10% glucose at different rates for clamping the level of blood glucose at 95 ± 5 mg/dl. **(B)** Changes of fasting plasma insulin. **(C)** Changes of blood glucose levels in ITT. **(D)** Values of AUC-ITT. E, Changes of blood glucose in IPGTT. **(F)** Values of AUC-IPGTT, *n* = 8. ^###^*p* < 0.001 vs. Con; ^*^p < 0.05, ^**^p < 0.01, ^***^p < 0.001 vs. DIO.

Glucose infusion rate (GIR) in hyperinsulinemic-euglycemic clamp test is the recognized gold index for the evaluation of insulin sensitivity *in vivo*. In our studies, the GIR value in DIO mice was 79.9% lower than that in Con mice. After 3 weeks of treatment, GIR was increased by 141.1, 158.7, and 108.7% in Rosi, Feno, and *Rho* group, respectively, compared with that in DIO group (Figure 3A).

Furthermore, to evaluate the insulin sensitizing effect of *Rho*, ITT, and IPGTT were conducted at day 22 and day 25, respectively. DIO mice exhibited a higher level of glucose in response to insulin and glucose load (Figures 3C,E). The two parameters, AUC-ITT (area under the curve in ITT) and AUC-IPGTT (area under the curve in IPGTT) were both much higher in DIO mice than those of control mice (Figures 3D,F). *Rho* administration suppressed the elevated glucose levels both in ITT and IPGTT (Figures 3C,E). Consistently, the elevated AUC-ITT and AUC-IPGTT was reduced by 18.9% and 15.5%, respectively (Figures 3D,F).

The FPI (fasting plasma insulin) was also determined to evaluate the insulin sensitizing effect of *Rho*. As shown in Figure 3B, DIO mice displayed notable hyperinsulinemia compared to Con mice; *Rho* treatment decreased the FPI significantly and had comparable effects with the positive drug.

### *Rho* ameliorates hepatic microcirculation disturbances in DIO mice

**Figure 4:**
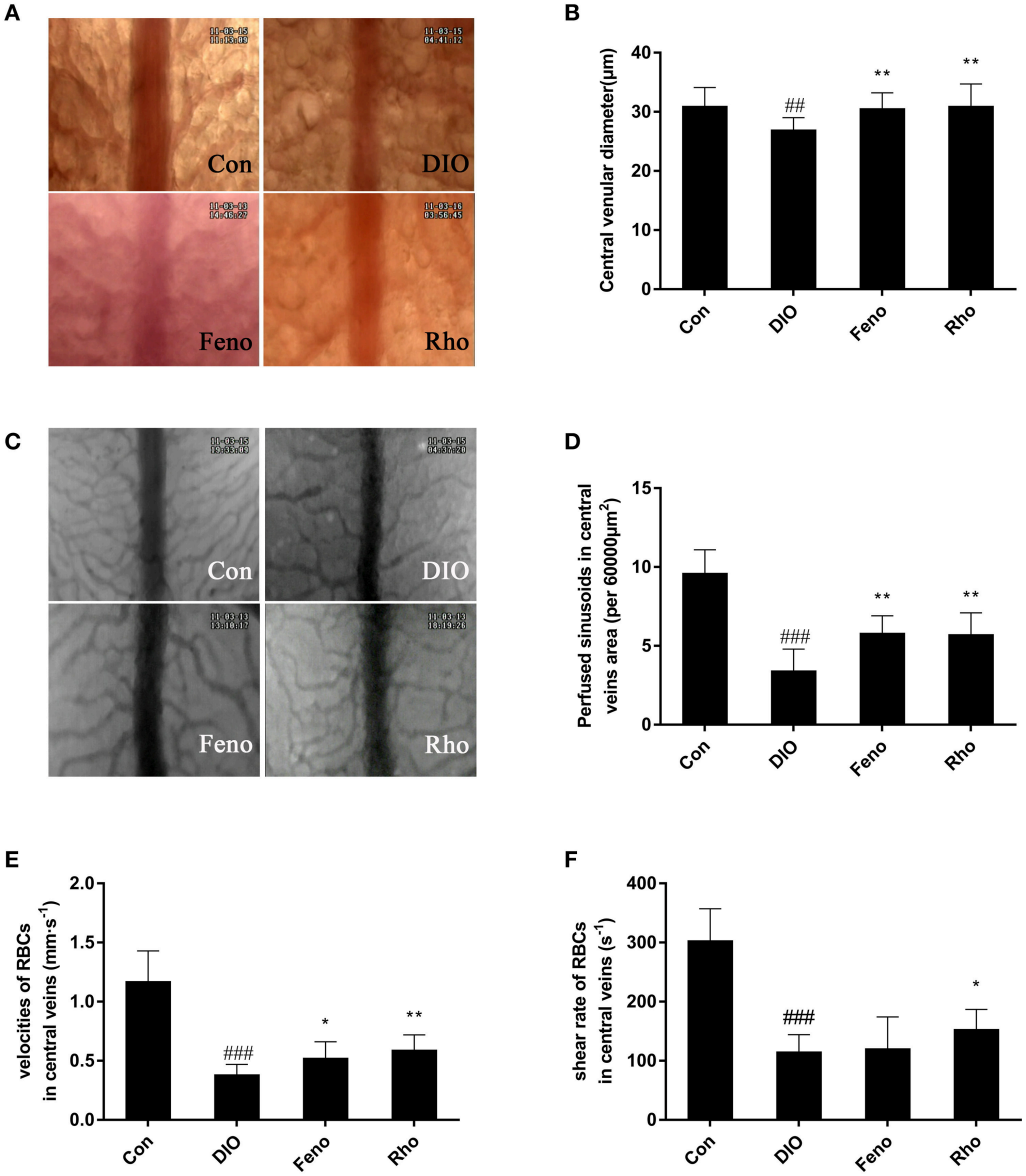
Effects of *Rho* on hepatic microcirculation disturbances in DIO mice. **(A)** Representative images of central venular diameter (×200). **(B)** Central venular diameter. **(C)** Representative images of sinusoids of central veins area (×200). **(D)** Perfused sinusoids in the central veins area (per field). **(E)** The velocity of RBCs in central veins. **(F)** Shear rates of RBCs in central veins. Data are shown as the means ± *SD*. ^##^*p* < 0.01, ^###^*p* < 0.001 vs. Con; ^*^p < 0.05, ^**^p < 0.01 vs. DIO, *n* = 8.

The hepatic microcirculation was observed by inverted microscopy, the central venular diameter, sinusoids perfusion, velocities of RBCs and shear rate of RBCs in central veins area were estimated, respectively. As shown in Figure 4, *Rho* could ameliorate the hepatic microcirculation disturbances. The narrowed central vein diameter in DIO mice was expanded by 14.9% after *Rho* treatment (Figures 4A,B). Compared with Con mice, the number of perfused sinusoids in central veins area was 64.6% decreased in DIO mice; after *Rho* treatment it was increased by 67.6% compared with that in DIO mice (Figures 4C,D). The RBCs velocity and share rates in central veins area were also enhanced by *Rho* treatment, and the enhancement was 55.3 and 32.9% compared with that in DIO mice, respectively (Figures 4E,F).

### *Rho* changes pathways involved in hepatic lipid metabolism in DIO mice

**Figure 5:**
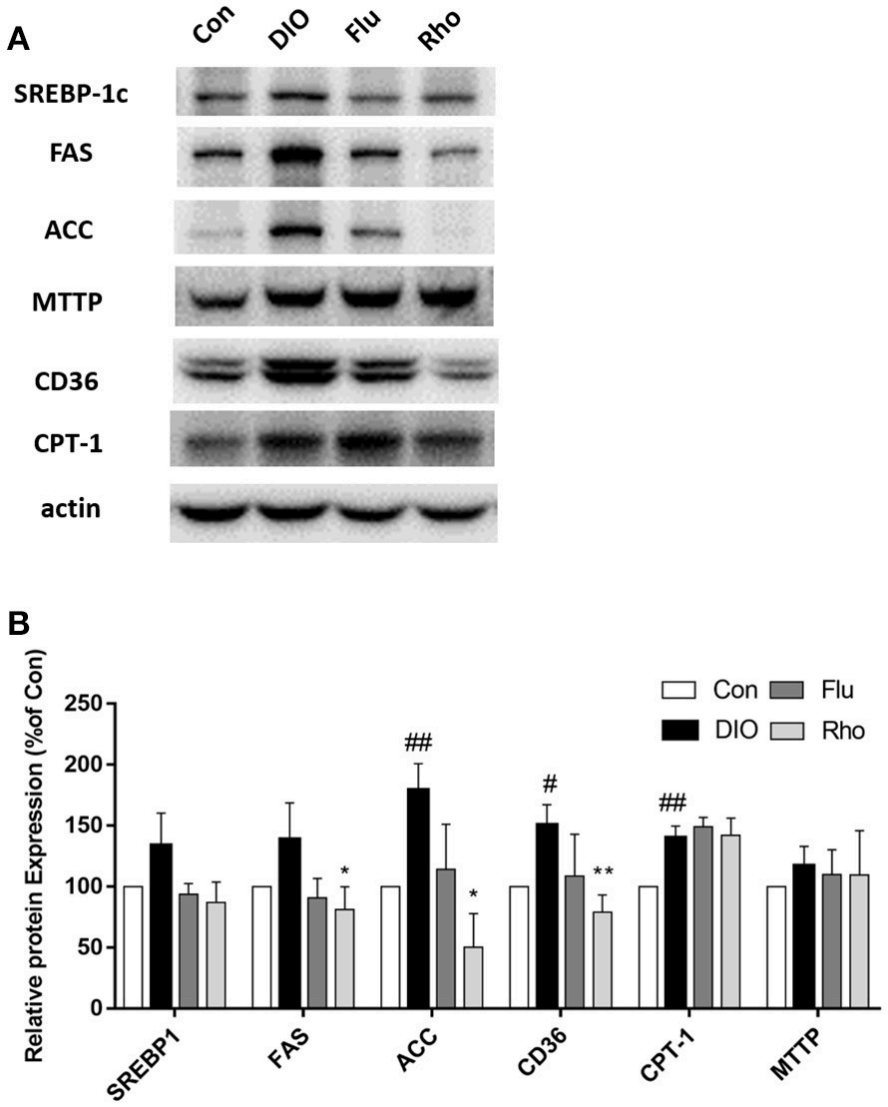
Effect of *Rho* on the pathways involved in hepatic lipid metabolism in DIO mice. **(A)** Expression of CD36, SREBP-1, FAS, ACC, CPT-1, and MTTP in liver. **(B)** Band intensities of proteins quantified by densitometry. Each data is from three independent tests. ^#^*p* < 0.05, ^##^*p* < 0.01 vs. Con; ^*^p < 0.05, ^**^p < 0.01 vs. DIO.

After 5 weeks of *Rho* treatment in DIO mice, the pathways involved in hepatic lipid metabolism including uptake, lipogenesis, oxidation and export were evaluated by Western Blot. As shown in Figure 5, the expression of CD36, SREBP-1c, FAS, ACC, CPT-1, and MTTP was up-regulated in livers of DIO mice, which indicated that the 4 pathways were all enhanced by HFD inducement. Remarkably, the pathways involved in fatty acid uptake and *de novo* lipogenesis were both down-regulated by *Rho* treatment with decreased expression of CD36, FAS, and ACC. However, the pathways involved in beta-oxidation and VLDL-export on hepatic steatosis were not changed significantly without changing the protein level of CPT-1 and MTTP.

## Discussion

Numerous studies have shown that *R. crenulata* has neuroprotective, anti-tumor, anti-inflammatory, anti-depression, anti-fatigue, immune regulation, and other effects (Lekomtseva et al., 2017; Wei et al., 2017). Lin et al. indicated that *R. crenulata* root extract (RCE), which contained 3.5% salidroside, regulated hepatic glycogen, and lipid metabolism *in vitro* (HepG2 cells) and *in vivo* (SD rats) via activation of the AMPK pathway (Lin et al., 2016). These results suggest that RCE is a potential intervention for patients with NAFLD. However, this inference is not reliable because the animal model used in this study did not have lipid accumulation in the liver, and the SD rats were fed RCE just for 3 days. David Vauzour et al. indicated that n-3 Fatty acids combined with flavan-3-ols (FLAV) prevent steatosis and liver injury in a murine model by regulating the expression of genes involved in hepatic lipid accumulation, such as PPARα (Vauzour et al., 2018). FLAV, a class of plant bioactive flavonoid compounds found in cocoa, tea, and berries, were found to have the effects of insulin sensitizing, antioxidant and anti-inflammation, and used as an emerging dietary strategy for NAFLD prevention (Rodriguez-Ramiro et al., 2016). In our study, the HPLC analysis indicated that the main components of *Rho* were flavan polymers. *Rho* ameliorated hepatic steatosis in three NAFLD mice models including high-fat diet-induced DIO mice, KKAy mice, and HFD combined with tetracycline stimulated Model-T mice. These results suggest that *Rho* is a lead nature product for NAFLD treatment.

### *Rho* decreased the uptake of free fatty acids via down-regulating the expression of CD36 in the liver of DIO mice

In steatosis, the early stadge of NAFLD, the imbalance between lipid storage and lipid removal in the liver results in the triacylglycerols (TAGs) accumulation (Bugianesi et al., 2010). The lipid storage mainly derived from the uptake of free fatty acids and *de novo* synthesis within the liver. The lipid removal is mainly derived from fatty acid oxidation in the mitochondria and the VLDL export. CD36 is an important membrane protein for FFAs uptake. The up-regulated expression of CD36 was observed in some pathological conditions such as obesity, diabetes and non-alcoholic fatty liver disease, resulted in the increasing uptake of free fatty acids into the liver (Koonen et al., 2007). Miquilena-Colina ME et al. showed that overexpression of CD36 is strongly associated with insulin resistance (Miquilena-Colina et al., 2011). In this study, the hepatic expression of CD36 in DIO mice was raised. *Rho* treatment reduced the expression of CD36, thus reduced the intake of liver fatty acids and improved lipid accumulation in the liver. Though the detail mechanism between insulin resistance and CD36 expression is still unknown, the down-regulation of CD36 might be relate to the improvement of insulin resistance. Carnitine palmitoyl transferase 1 (CPT1) is the key factor in fatty acid oxidation in the mitochondria (Orellana-Gavaldà et al., 2011). The human microsomal triglyceride transfer protein (MTTP) carries lipid transfer function to remove lipid from liver by the assembly and secretion of very-low-density lipoprotein (VLDL) (Pereira et al., 2011). In DIO mice, the CPT1 expression was up-regulated, and there were no changes observed in the MTTP expression. *Rho* treatment did not affect the expression of the two proteins mentioned above. These results indicated that the pathways involved in beta-oxidation and VLDL-export on hepatic steatosis were not changed significantly after *Rho* treatment. Usually, hepatic lipogenesis means TG synthesized from the esterification of free fatty acids (FFA) with glycerol-3-phosphate (Rodríguez et al., 2006). Tglycerol-3-phosphate derives from three metabolic sources: (1) glucose from glycolysis; (2) lipolysis-derived glycerol; and (3) glycerol uptake mediated by AQP9 (Rodríguez et al., 2011; Calamita et al., 2012), which expresses lower during NAFLD in patients and in murine models (Gena et al., 2013; Rodríguez et al., 2014). The down-regulated expression of AQP9 may be a compensatory mechanism to decrease the *de novo* TG synthesis. Sexual dimorphism in hepatocyte glycerol permeability might be a significant cause for their different prevalence of insulin resistance and NAFLD (Rodríguez et al., 2015). The change of the AQP9 and the gender difference was not observed in this study. These issues should be addressed in the future.

### *Rho* decreased the *de Novo* lipogenesis via down-regulating the expression of SREBP-1c, FAS, and ACC in the liver of DIO mice

The expressions of SREBP-1c, FAS, and ACC in the liver affect the hepatic *de novo* synthesis. SREBP-1c is considered the major mediator for the insulin-regulated lipogenesis. It is up-regulated in NAFLD and plays an important role in the lipid accumulation in fatty liver (Ferre and Foufelle, 2010). In our study, the hepatic expression of SREBP-1c in the DIO mice was significantly higher than that in Con mice. *Rho* had a tendency to reduce SREBP-1c expression in DIO mice. SREBP-1c has been reported to activate FAS and ACC promoters (Wang et al., 2015). Fatty acid synthase (FAS) can catalyzes the *de novo* synthesis of fatty acids in cytoplasm (Jensen-Urstad and Semenkovich, 2012). Our results showed that the hepatic expression of FAS in DIO mouse was significantly higher than that in Con mice; *Rho* has the effect of reducing FAS protein expression, reducing *de novo* synthesis of fatty acids in liver to improve lipid accumulation in the liver. The first step, also the rate limiting step, in fatty acid biosynthesis is the reaction of ATP-dependent carboxylation of acetyl-CoA to form malonyl-CoA, and is catalyzed by acetyl-CoA carboxylase (ACC) (Tong and Harwood, 2006). In this study, the hepatic expression of ACC in DIO mice was significantly higher than that in Con mice. *Rho* has the effect of down-regulating ACC protein expression and reducing the synthesis of fatty acids.

### *Rho* decreased the *de Novo* lipogenesis via improving insulin resistance in DIO mice

Insulin resistance plays a key role in hepatic lipid (especially fatty acids) accumulation and the subsequent increase of adipose tissue lipolysis (Bugianesi et al., 2010). In insulin resistance state, the pancreas compensates to increase the production of insulin to maintain normal glucose levels. It is reported that the higher level of insulin over-stimulates *de novo* lipogenesis, and leads to lipids accumulation based on the regulation of SREBP-1c (Shimomura et al., 2000; Konner and Bruning, 2012). As mentioned above, SREBP-1c is a key regulator in *de novo* adipogenesis (Rawson, 2003; Wang et al., 2015). Insulin is considered an important SREBP-1c activator that induces SREBP-1c expression via multiple insulin signaling pathways such as mTORC1, PI3K-AKT, and others (Wong and Sul, 2010; Shao and Espenshade, 2012; Alam et al., 2016). This indicates that improving insulin resistance may reduce the lipid synthesis and accumulation in the liver. This insulin sensitizing effect may relate to the down-regulated SREBP-1c expressions in *Rho* treatment group.

### *Rho* improved insulin resistance via inhibiting PTP1B activity in DIO mice

The protein tyrosine phosphatase1B (PTP1B) is a negative regulator of both insulin and leptin signaling, and shows a highly validated therapeutic target for the treatment of diabetes and obesity (Zhang and Zhang, 2007). In previous study in our lab, *Rho* was proved to have inhibitory effect on PTP1B (IC50 = 0.106 mg·L^−1^) (Tian et al., 2016). This might contribute to the insulin sensitizing effects of *Rho*. The mechanism of the improvement effects of Rho was summarized as follows: 
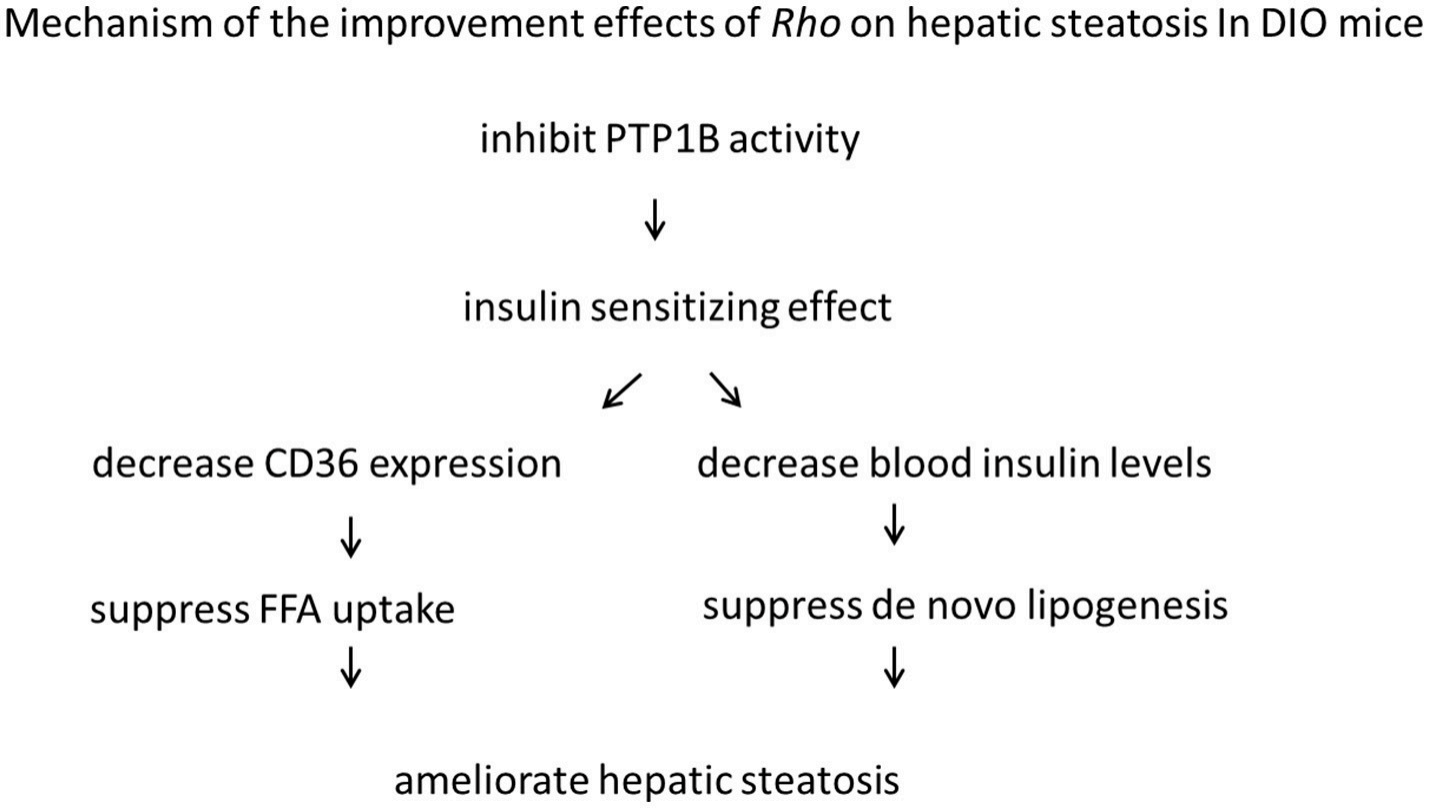


Furthermore, *Rho* was observed to ameliorate hepatic microcirculation disturbances in DIO mice in this study. It might be speculated that *Rho* ameliorated the lipid accumulation in liver, and the specific mechanism needs to be further explored.

In conclusion, our data suggest that *Rho* is a lead nature product for hepatic steatosis treatment. Its mechanism is related to improving insulin resistance, suppressing fatty acid uptake, inhibiting *de novo* lipogenesis, and could ameliorate microcirculatory disturbances in liver.

## Author contributions

QY, PS, SK, JT, and XL conducted the pharmacological experiments and performed data analysis; YY and PZ prepared the active fractions from *Rhodiola crenulata* (Rho); JL, YL, and JH designed and conducted the experiments in hepatic microcirculation observation; XZ and FY designed the study, made data interpretation and prepared the manuscript.

### Conflict of interest statement

The authors declare that the research was conducted in the absence of any commercial or financial relationships that could be construed as a potential conflict of interest.
